# White‐tailed deer detection rates increase when coyotes are present

**DOI:** 10.1002/ece3.11149

**Published:** 2024-03-17

**Authors:** Hannah L. Clipp, Sarah M. Pesi, Madison L. Miller, Laura C. Gigliotti, Brett P. Skelly, Christopher T. Rota

**Affiliations:** ^1^ Division of Forestry and Natural Resources West Virginia University Morgantown West Virginia USA; ^2^ West Virginia Cooperative Fish and Wildlife Research Unit West Virginia University Morgantown West Virginia USA; ^3^ U.S. Geological Survey West Virginia Cooperative Fish and Wildlife Research Unit Morgantown West Virginia USA; ^4^ West Virginia Division of Natural Resources Elkins West Virginia USA

**Keywords:** camera trap, coyote, detection rate, multi‐species occupancy model, predator–prey dynamics, white‐tailed deer

## Abstract

Predator species can indirectly affect prey species through the cost of anti‐predator behavior responses, which may involve shifts in occupancy, space use, or movement. Quantifying the various strategies implemented by prey species to avoid adverse interactions with predators can lead to a better understanding of potential population‐level repercussions. Therefore, the purpose of this study was to examine predator–prey interactions by quantifying the effect of predator species presence on detection rates of prey species, using coyotes (*Canis latrans*) and white‐tailed deer (*Odocoileus virginianus*) in Central Appalachian forests of the eastern United States as a model predator–prey system. To test two competing hypotheses related to interspecific interactions, we modeled species detections from 319 camera traps with a two‐species occupancy model that incorporated a continuous‐time detection process. We found that white‐tailed deer occupancy was independent of coyote occupancy, but white‐tailed deer were more frequently detectable and had greater detection intensity at sites where coyotes were present, regardless of vegetation‐related covariates. In addition, white‐tailed deer detection rates at sites with coyotes were highest when presumed forage availability was relatively low. These findings suggest that white‐tailed deer may be exhibiting an active avoidance behavioral response to predators by increasing movement rates when coyotes are present in an area, perhaps due to reactive evasive maneuvers and/or proactive attempts to reduce adverse encounters with them. Concurrently, coyotes could be occupying sites with higher white‐tailed deer densities. Because white‐tailed deer did not exhibit significant shifts in daily activity patterns based on coyote occupancy, we further suggest that white‐tailed deer in our study system generally do not use temporal partitioning as their primary strategy for avoiding encounters with coyotes. Overall, our study implements a recently developed analytical approach for modeling multi‐species occupancy from camera traps and provides novel ecological insight into the complex relationships between predator and prey species.

## Graphical abstract

In this study, we quantified the effect of predator species presence on camera trap detection rates of prey species, using coyotes (*Canis latrans*) and white‐tailed deer (*Odocoileus virginianus*) in Central Appalachian forests of the eastern United States as a model predator–prey system. We tested two competing hypotheses related to predator–prey interactions by modeling detections of each species from 319 camera traps with a two‐species occupancy model that incorporated a continuous‐time detection process. We found that white‐tailed deer had higher detection rates at sites where coyotes were present, suggesting that white‐tailed deer may be exhibiting an active avoidance behavioral response to predators by increasing movement rates when coyotes are present in an area; concurrently, coyotes could be occupying sites with higher white‐tailed deer densities.

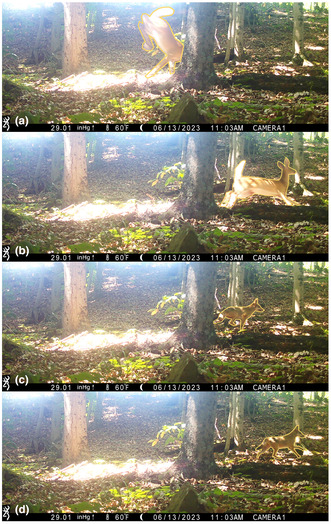

## 1 INTRODUCTION

Predators can affect prey populations both directly, by inflicting injury or causing mortality to individuals (i.e., predation), and indirectly through the cost of anti‐predator behavior responses (Creel & Christianson, 2008). Prey species may alter their behavior in several ways when responding to the presence of predators. For example, individuals might increase vigilance behaviors (Creel et al., 2017; Lima, 1987) when they perceive predation risk to be high. Prey species may also seek out refuge, utilize cover at increased rates, or shift locations when predators are present (Fraser & Huntingford, 1986). In concordance, individuals are likely to modify their occupancy and space use patterns when under the risk of predation by preferentially selecting safe habitats and avoiding areas where they are more likely to encounter predators (Thaker et al., 2011). Ultimately, when predators are present in the landscape, changes in the behavioral responses of prey species can consequently influence survival probability and reproductive success by increasing energetic or physiological costs (DeCesare et al., 2014; Zanette et al., 2011). Quantifying the various strategies implemented by prey species to avoid adverse interactions with predators can therefore potentially lead to a better understanding of prey population dynamics. Altered prey behaviors enacted to reduce predation risk may also have repercussions for the larger ecosystem by causing trophic cascades (Ford et al., 2014; Suraci et al., 2016).

The presence of predators on the landscape can influence multiple spatiotemporal components pertaining to occupancy, spatial ecology, and movement patterns of prey species. Prey might completely avoid areas with a high perceived risk of predation (Laundré et al., 2014; Lima & Dill, 1990), or restrict their use of high‐risk areas to time periods when predators are less active (Frey et al., 2017). Alternatively, rather than avoiding risky areas, individuals might vary their movement rates (i.e., frequency of ambulatory or nonstationary movement) with respect to the level of predation risk. For example, prey species may exhibit increased movement rates in high‐risk areas to reduce the probability of encountering predators (Frair et al., 2005; Sih, 1984). Increasing movement rates can be especially beneficial for prey species in systems where predator species have good spatial memories (Mitchell & Lima, 2002). Another strategy would be for prey to decrease movement rates in high‐risk areas when habitat features (e.g., vegetation structure) facilitate cover and concealment, such that reduced movement rates would decrease detection by or encounter rates with predators (Griffin et al., 2005; Smith, Donadio, Pauli, Sheriff, & Middleton, 2019).

Previous research on the effects of predators on the spatial ecology of prey species has typically relied on animal location data obtained from GPS or VHF transmitters, which can be used to assess overlap in occupancy, space use, and/or encounter rates between predators and prey. However, these data can be costly and time‐intensive to collect (Suraci et al., 2022). As an alternative to transmitter location data, camera traps can be a cost‐effective and noninvasive method used to collect image data of predator and prey species. Previous camera trap research has investigated various effects of predators on prey occurrence and space use, including analyses of temporal overlap patterns (Ridout & Linkie, 2009), spatiotemporal patterns (Cusack et al., 2016; Niedballa et al., 2019), or multi‐species occupancy (Rota et al., 2016). Recently, a two‐species occupancy model with a continuous detection process (Kellner et al., 2022) was developed to allow for the estimation of spatial and temporal interactions of two species while accounting for imperfect detection. This type of analytical approach facilitates modeling variation in prey species detection rates in the presence vs. absence of predator species. A difference in detection rates could be attributed to changes in either movement rates (Popescu et al., 2014) or population densities (Parsons et al., 2017), such that increased detection rates of a prey species when a predator species is present could result from prey responding to predators by increasing movement rates or predators responding to higher prey population densities.

With the novel two‐species occupancy model incorporating a continuous‐time detection process (Kellner et al., 2022), we studied the effects of predator species presence on detection rates of prey species, using coyotes (*Canis latrans*) and white‐tailed deer (*Odocoileus virginianus*) in Central Appalachian forests of the eastern United States of America (USA) as a model predator–prey system. In North America, coyotes act as apex predators in ecosystems when larger carnivores are absent (Gompper, 2002), and frequently prey upon neonates (Hinton et al., 2021; Kilgo et al., 2012). There are cases where coyotes limited the population growth of white‐tailed deer through fawn mortality (Kilgo et al., 2012), although a recent study found that fawn survival in sites in the state of Delaware (Mid‐Atlantic Coast of the eastern USA) with no predator species was similar to areas with established predator communities (Dion et al., 2020). Thus, the role of coyote predation on white‐tailed deer population dynamics is still highly contested, but it may be mediated by ecological carrying capacity, such that the impact of predation is limited when white‐tailed deer populations are at or near carrying capacity (Ballard et al., 2001).

Previous research indicates that white‐tailed deer exhibit behavioral changes in response to the presence or abundance of coyotes. For example, increased coyote abundance is associated with decreased white‐tailed deer foraging behavior as they allocate more time to vigilance (Gulsby et al., 2018). Additionally, coyotes influence movement patterns of white‐tailed deer, resulting in both spatial and temporal modifications. Studies have found that white‐tailed deer consistently moved down and away from sloped landscapes in the presence of coyotes (Lingle, 2002), and maternal females with fawns significantly changed their activity patterns from crepuscular to mid‐day activity to not overlap with the crepuscular or nocturnal activity of coyotes (Higdon et al., 2019). When encounters with coyotes do occur, white‐tailed deer generally exhibit a fleeing response to coyote pursuit (Grovenburg et al., 2012; Lingle & Pellis, 2002). Because coyotes have documented effects on white‐tailed deer behavior, coupled with their wide distributions across North America, they serve as an ideal and relevant study system to deepen our understanding of predator–prey interactions.

**FIGURE 1:**
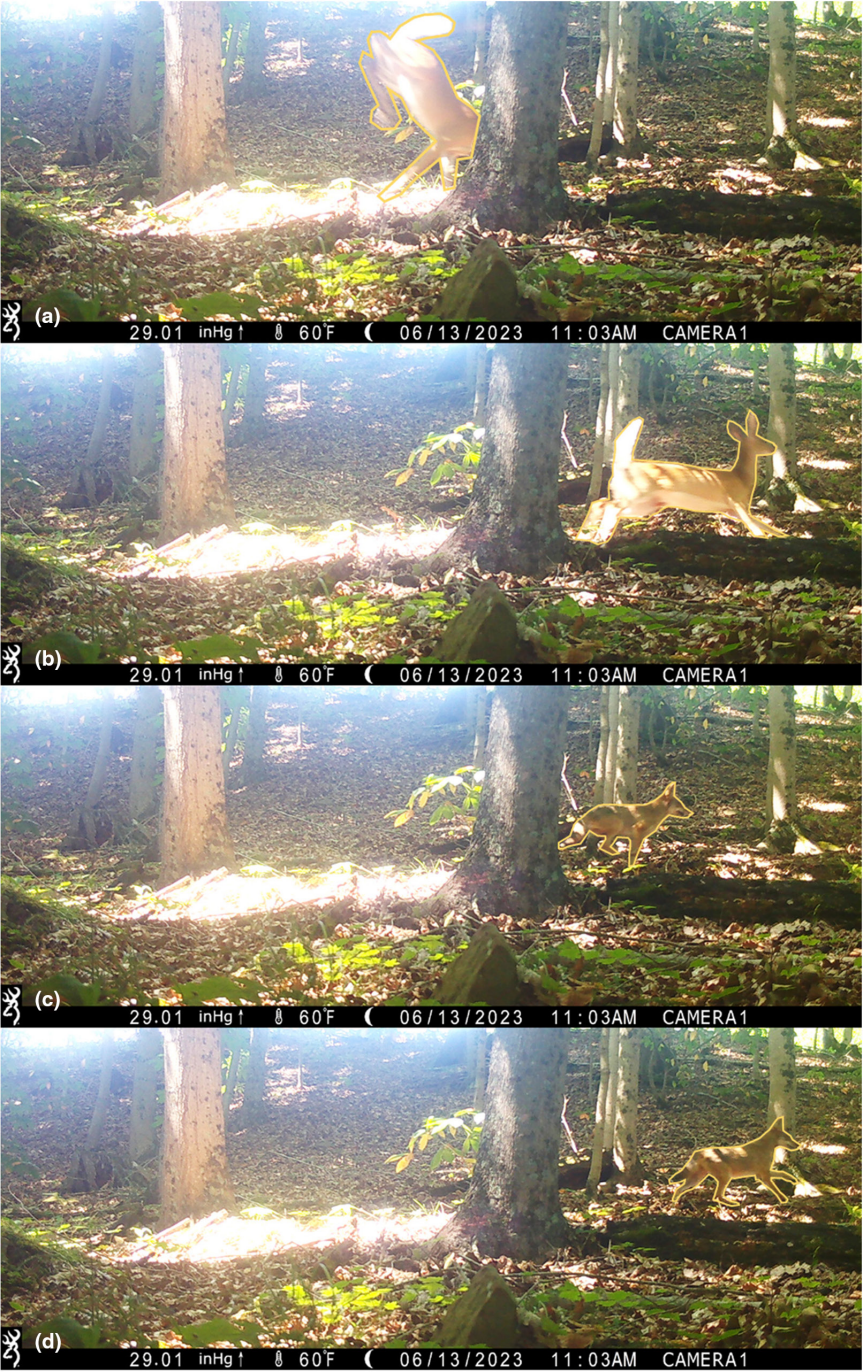
Sequence of four camera trap images of the focal study species, showing an adult white‐tailed deer (*Odocoileus virginianus*; a, b) fleeing from a coyote (*Canis latrans*; c, d). All four images of this diurnal pursuit event were captured by a single camera trap in the displayed order within the same minute (11:03 AM). Yellow outlines were manually added as visual aids.

Using an extensive dataset of coyote and white‐tailed deer detections (i.e., photographs; Figure 1) from 319 camera traps deployed as part of three independent research studies, we modeled occupancy and detection rates of each species, and tested two competing hypotheses related to interactions between predator and prey species:

1. Active Response Hypothesis, which posits that white‐tailed deer would exhibit increased detection rates when coyotes were present because more movement would result from fleeing behavior (i.e., reactive response) and/or decrease the probability of adverse encounters with coyotes (i.e., proactive response), and/or because coyotes tended to occupy sites with higher white‐tailed deer densities.
2. Predation Refuge Hypothesis, which posits that white‐tailed deer would exhibit decreased detection rates when coyotes were present because remaining localized in a “safe” area (resulting in decreased movement rates) would lessen the probability of encounters with coyotes, and/or because white‐tailed deer might have occurred at lower densities in areas with coyotes due to intentional avoidance or mortality.

The main objective of this study was to quantify how coyotes influence detection rates of white‐tailed deer in forested landscapes within the Central Appalachians and potentially across eastern North America. Although previous investigations have shown that white‐tailed deer alter some vigilance and movement behaviors in the presence of coyotes, our study aims to determine whether camera trap detection rates of prey species increase or decrease in areas where predators are present. Overall, we seek to elucidate predator–prey interactions and understand the mechanisms that may be driving variation in detection rates of prey species in response to predator species.

## 2 METHODS

### 2.1 Study area

**FIGURE 2:**
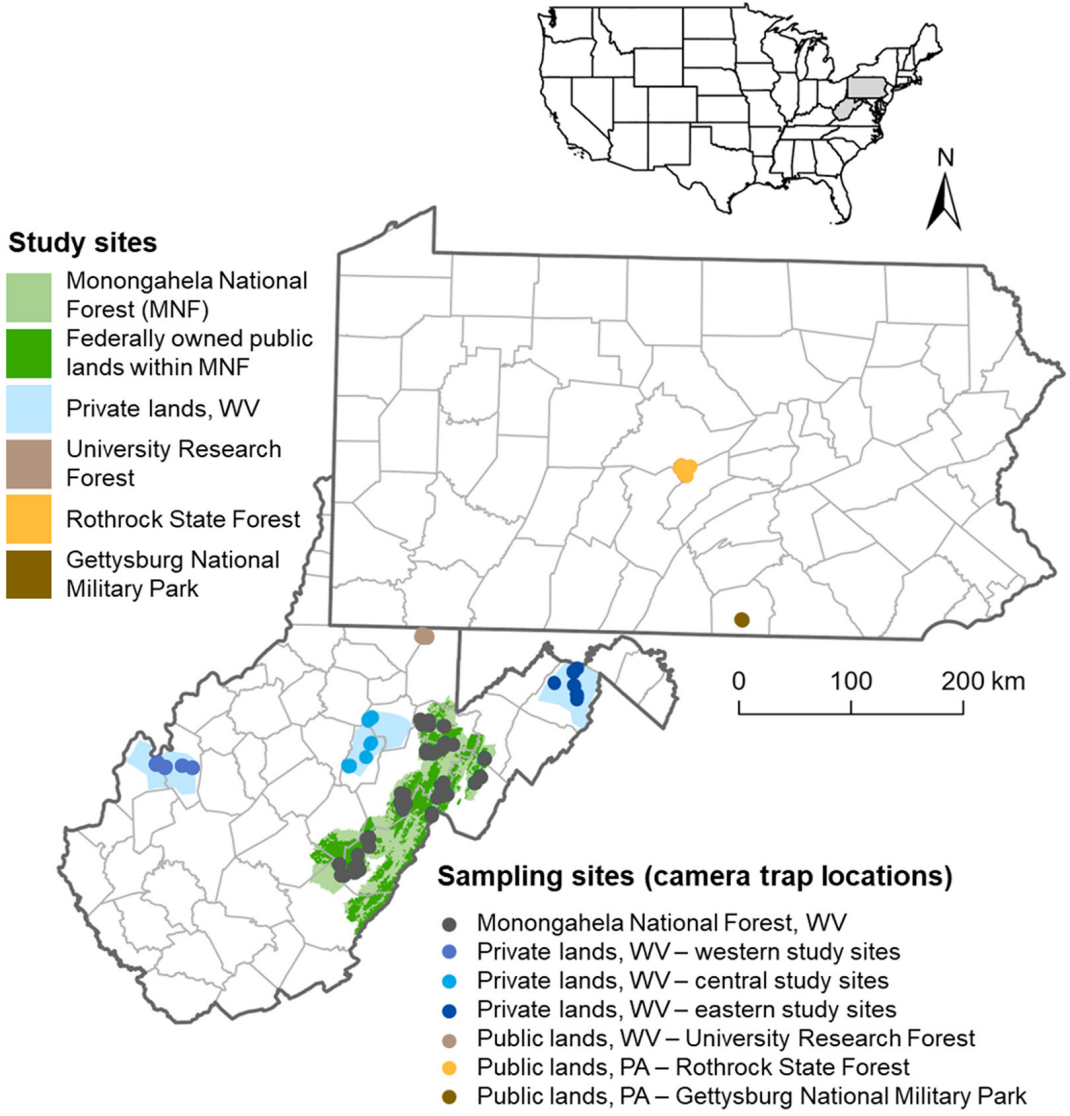
Locations of 319 camera traps throughout the Central Appalachians region (West Virginia and Pennsylvania) of the eastern USA. Data were compiled from three independent research studies, consisting of seven primary study sites: Monongahela National Forest (where camera traps were deployed in April–May 2019–2021 at a total of 129 wildlife openings); private landowner properties (where camera traps were deployed in April–September 2022 at a total of 138 locations) across five counties of West Virginia; and public forested lands (where camera traps were deployed in August–November 2020 at a total of 52 locations) in West Virginia and Pennsylvania, as part of Snapshot USA 2020.

Camera trap data for this investigation were obtained from three independent research studies, with sampling sites located throughout the Central Appalachians region (Figure 2). Coyotes, along with American black bears (*Ursus americanus*) and bobcats (*Lynx rufus*), are the primary natural predators of white‐tailed deer in this region. The first dataset consisted of 129 camera traps deployed during 2019–2021 in wildlife openings within the Monongahela National Forest (MNF) in West Virginia; the second dataset consisted of 138 camera traps deployed during 2022 on private landowner properties in three study sites across five counties of West Virginia; and the third dataset consisted of 52 camera traps deployed in 2020 on public forested lands at one study site in West Virginia and at two study sites in Pennsylvania as part of Snapshot USA 2020, a coordinated camera trap survey across the USA (Kays et al., 2022). Altogether, our seven study sites span multiple physiographic provinces and several different forest types that are representative of Central Appalachia.

#### 2.1.1 Monongahela National Forest

The MNF encompasses portions of nine counties (Preston, Tucker, Grant, Randolph, Pendleton, Pocahontas, Webster, Nicholas, and Greenbrier) in eastern West Virginia. Established in 1920, the full extent of the national forest stretches across a latitudinal range of nearly 200 km and covers nearly 688,000 ha, with federally owned public lands comprising 54% (372,715 ha). Much of the MNF is located in the Allegheny Mountain section of the Appalachian Plateau physiographic province, with the eastern portion of the national forest within the Ridge and Valley physiographic province. Elevation within the MNF ranges from 275 to 1480 m. The MNF primarily consists of 70–100 years old stands with high levels of regional tree diversity and four major forest zones (mixed mesophytic, northern hardwoods, red spruce [*Picea rubens*], and dry oaks [*Quercus* spp.]), corresponding to gradients in elevation and precipitation. There are >2200 wildlife openings (i.e., areas of early‐successional vegetation, including herbaceous, shrubby, and young forest habitat, that are surrounded by mature forest) located throughout the public lands of the MNF. The wildlife openings include natural forest openings and those that were created purposefully for target wildlife species or as a by‐product of logging or mining, and they range in shape and size, from 0.009 to 113 ha, although the vast majority (96%) are <5 ha.

#### 2.1.2 Private lands in West Virginia

The data collected on private properties in West Virginia were from camera traps dispersed across five counties, located in Hampshire County within the eastern region of the state, in Barbour and Upshur Counties within the central region of the state, and in Jackson and Mason Counties within the western region of the state. All of the study sites contained <1.5% public lands and were >80% forested, with a dominant forest type of oak‐hickory. The eastern study site was located in the Ridge and Valley physiographic province, with elevation ranging from 160 to 780 m. The central and western study sites were located in the Appalachian Plateau physiographic province, with elevation ranging from 310 to 880 m and 160 to 370 m, respectively.

#### 2.1.3 Public lands in West Virginia and Pennsylvania

Snapshot USA 2020 data were collected from public lands in West Virginia and Pennsylvania (Figure 2), including the West Virginia University Research Forest (located on the borders of Monongalia and Preston Counties in West Virginia), Rothrock State Forest (spanning Huntingdon, Centre, and Mifflin Counties in Pennsylvania), and Gettysburg National Military Park (located in Adams County, Pennsylvania). The West Virginia University Research Forest encompasses ~3075 ha within the Allegheny Mountain section of the Appalachian Plateau physiographic province and is predominantly even‐aged as a result of heavy logging in the 1930s, consisting of 60–80 years old stands of mixed oak and mesophytic hardwood types. Located in the Ridge and Valley physiographic province, Rothrock State Forest comprises 39,244 ha and is dominated by oak‐hickory forests. Gettysburg National Military Park contains ~2430 ha within the Piedmont physiographic province and consists of oak‐hickory forests interspersed with roads and human structures.

### 2.2 Camera trap deployment

Sampling in the seven study sites was considered spatially and temporally independent. Spatial independence of sampling sites was ensured with minimum distances (≥200 m) between camera trap locations. The timing of camera trapping at specific sampling sites from the three independent research studies varied, but all camera traps were deployed for ≤76 days, which we deemed a reasonable amount of time to expect closure (i.e., no change in occupancy across the sampling period). Despite differences in the number and deployment length of camera traps, they were deployed following similar set‐up methods (e.g., height from ground, orientation) across the seven study sites. In addition, all camera traps in the investigation were of comparable quality, were equipped with an infrared flash, and had trigger speeds of <0.5 s; the latter two features allowed the camera traps to be able to record animals passing in front of them without the need for baiting.

In the MNF, camera traps were deployed for 2–12 days (mean: 8.9 days) between April 13 and May 5, 2019–2021 at a total of 129 wildlife openings, which were selected through stratified random sampling based on size categories and maintenance status. Most wildlife openings tended to be relatively small (<1.5 ha) and generally round, with low edge‐to‐area ratios (<0.07), high percentages (100%) of herbaceous cover, and lower percentages of shrub (<25%), sapling (<10%), and tree cover (<20%). Nearly 40% of the wildlife openings were maintained by land managers, and while 33% of the wildlife openings were located within 50 m of a road, the majority (>75%) had no to low apparent human activity, based on qualitative field observations. From a landscape‐level context, the wildlife openings were distributed across the MNF's elevational gradient, and most had high percentages (>90%) of forest cover within 1 km. At each sampling site, a single camera trap (Bushnell Trophy Cam HD or Reconyx Hyperfire) was set up 40–50 cm above the ground, oriented parallel with the ground, and located at a randomly generated location <50 m from the center point of the wildlife opening. Each camera trap was set on maximum trigger sensitivity and recorded multiple photographs per trigger, re‐triggering immediately if the animal was still in view.

On private landowner properties in West Virginia, camera traps were deployed between April 23 and September 15, 2022 and recorded data for 5–64 days (mean: 59.4 days) at 41 locations in the eastern study site, 6–76 days (mean: 63 days) at 56 locations in the central study site, and 2–67 days (mean: 57.3 days) at 41 locations in the western study site. Camera trap placement was based on grids with 400 m spacing, and grid size ranged from 4 to 33 cameras. Camera traps were deployed on trees or fence posts 50 cm above the ground, oriented parallel with the ground and not facing directly east or west. They were set to take a 3‐photograph burst when triggered, with the minimum quiet time between triggers.

For the camera traps deployed as part of Snapshot USA 2020, participating scientists followed standardized protocols described in Kays et al. (2022). Between August 31 and November 5, 2020, camera traps were deployed for 2–43 days (mean: 22.3 days) at a total of 33 locations in the West Virginia University Research Forest, for 28–56 days (mean: 49.3 days) at a total of 12 locations in Rothrock State Forest, and for 2–50 days (mean: 29.4 days) at a total of seven locations in Gettysburg National Military Park. At each forested study site, camera trap arrangements were set up at the discretion of the Snapshot USA 2020 participants, but all camera traps at a study site were located at a minimum distance of 200 m and a maximum distance of 5 km from neighboring camera traps. Each camera trap was placed 50 cm off the ground, with their orientation parallel to the slope.

Variation in the deployment length of individual camera traps and temporal differences in the overall sampling periods among the three independent research studies limited our ability to explicitly model season and white‐tailed deer age classes as potential explanatory covariates. However, we did account for seasonality and site‐specific variation in vegetation and forage availability by using measures of the Normalized Difference Vegetation Index (NDVI; Pettorelli et al., 2011). For each study site, we used 30‐m resolution rasters of mean NDVI during the periods of camera trap deployment and extracted NDVI values for each individual camera trap location.

### 2.3 Camera trap data processing

Camera trap images were processed using either eMammal (MNF and Snapshot USA 2020) or Wildlife Insights (private lands in West Virginia) applications. Both applications grouped photographs into sequences based on the time elapsed between photographs. A single sequence was comprised of an ordered series of photographs where successive images were taken within 1 min of each other. Note that the duration of a single sequence could be greater than 1 min as long as the time between each successive photograph was <1 min. Trained data processing assistants recorded the species detected within each photograph sequence. For data analysis, we extracted the starting timestamp and deployment identification of all sequences containing coyotes and/or white‐tailed deer.

### 2.4 Data analyses

We used multi‐species occupancy models with continuous‐time detection processes to test for spatial and temporal interactions between coyotes and white‐tailed deer. This model is an extension of Rota et al.'s (2016) multi‐species occupancy model and permits us to test whether species occur independently in space. We therefore assume the latent presence/absence of coyotes and white‐tailed deer at sampling site *i* is a multivariate Bernoulli random variable:

$$Z_{i}\sim \text{Multivariate Bernoulli}\left(\Psi_{i}\right),$$

where ***Z***_*i*_ is a vector of length two denoting latent presence/absence of coyotes and white‐tailed deer and ***Ψ***_*i*_ is a vector of length four describing the probability of all four possible latent states. The vector ***Ψ***_*i*_ is calculated as a function of parameters that describe the log odds of each species occurring when the other is absent (*f*_1_ and *f*_2_), and the change in log odds that one species occurs at a sampling site when the other is present (*f*_3_):

$$\psi_{11}=\frac{\exp\left(f_{1}+f_{2}+f_{3}\right)}{1+\exp\left(f_{1}\right)+\exp\left(f_{2}\right)+\exp\left(f_{1}+f_{2}+f_{3}\right)},$$

$$\psi_{10}=\frac{\exp\left(f_{1}\right)}{1+\exp\left(f_{1}\right)+\exp\left(f_{2}\right)+\exp\left(f_{1}+f_{2}+f_{3}\right)},$$

$$\psi_{01}=\frac{\exp\left(f_{2}\right)}{1+\exp\left(f_{1}\right)+\exp\left(f_{2}\right)+\exp\left(f_{1}+f_{2}+f_{3}\right)},$$

$$\psi_{01}=\frac{1}{1+\exp\left(f_{1}\right)+\exp\left(f_{2}\right)+\exp\left(f_{1}+f_{2}+f_{3}\right)}.$$

Parameters *f*_1_, *f*_2_, and *f*_3_ can be modeled as linear functions of covariates.

We then use a Markov‐modulated point process (MMPP) to model detections in continuous time. Detection rates (i.e., the number of photographs per unit of time) can be considered a function of two processes: availability for detection, and detection intensity when available for detection. The MMPP allows us to parse these two processes by permitting species to switch between “detectable” and “non‐detectable” states, and by estimating detection intensity when in the “detectable” state. In this context, a species is in the “detectable” state if it is close to a camera trap and available to be photographed. A species is in the “non‐detectable” state if it is present at a sampling site but unavailable for detection, perhaps because it is not in the immediate area of a camera trap. Detection intensity is then defined as the number of photographs per unit of time when the species is available to be detected. The MMPP likelihood is based on a vector of time intervals Δ_*si*_ between detections of species *s* at sampling site *i* as follows:

$$L\left(\Delta_{\mathrm{si}}|z_{\mathrm{si}}=1\right)=\pi_{s}\left(\prod_{j=1}^{n_{\mathrm{si}}}\exp\left\{\left(Q_{s}-\Lambda_{s}\right)\delta_{\mathrm{sij}}\right\}\Lambda_{s}\right)\exp\left\{\left(Q_{s}-\Lambda_{s}\right)\left(T_{i}-t_{\mathrm{si}n_{\mathrm{si}}}\right)\right\}e,$$

where ***Q***_*s*_ is a matrix describing how long species *s* remains within each state before switching to the other state and ***Λ***_*s*_ is a matrix describing detection intensity within each state. Additional model variables include *π*_*s*_, which is a vector describing initial probability of being in either state; *n*_*si*_, which is the total number of detections of species *s* at site *i*; *δ*_*sij*_, which is the time interval between detection *j* and the previous detection; *T*_*i*_, which is the total time the detector was deployed; and $t_{\mathrm{si}n_{\mathrm{si}}}$, which is the time of the last detection of species *s* at sampling site *i*. When a species is not detected at a sampling site, the likelihood collapses to:

$$L\left(\Delta_{\mathrm{si}}|z_{\mathrm{si}}=1\right)=\pi_{s}\exp\left\{\left(Q_{s}-\Lambda_{s}\right)\left(T_{i}\right)\right\}e.$$

Matrix ***Q***_*s*_ is composed of rate parameters exp(*μ*_*s*1_) and exp(*μ*_*s*2_) that define how long each species stays in each detectable state:

$$Q_{s}=\left[-\exp\left(\mu_{s1}\right)\;\exp\left(\mu_{s1}\right)\;\exp\left(\mu_{s2}\right)-\exp\left(\mu_{s2}\right)\;\right].$$

Similarly, matrix ***Λ***_*s*_ is composed of rate parameter exp(*λ*_*s*_) that describes the detection intensity:

$$\Lambda_{s}=\left[0\;0\;0\;\exp\left(\lambda_{s}\right)\;\right].$$

Detection intensity in each of the two states is represented along the matrix diagonal. By forcing detection intensity in one of the two states to 0, we create a “detectable” and “non‐detectable” state. Parameters *μ*_*s*1,_ *μ*_*s*2_, and *λ*_*s*_ can all be modeled as functions of covariates. The MMPP can also accommodate different deployment lengths for each camera trap. See Kellner et al. (2022) for additional details.

We evaluated interactions by testing if (1) white‐tailed deer and coyote site occupancy were independent; (2) the proportion of time that a white‐tailed deer was in the detectable state varied at sites with and without coyotes; and (3) the detection intensity of white‐tailed deer while in the detectable state varied at sites with and without coyotes. We evaluated whether white‐tailed deer and coyotes occurred independently by testing if interaction parameter *f*_3_ was different from 0 (assuming a 0.05 type‐1 error probability). We otherwise assumed white‐tailed deer and coyote interactions were constant across space (i.e., we did not model parameter *f*_3_ as a function of covariates).

We evaluated the proportion of time that white‐tailed deer (wtd) spent in the detectable state by allowing matrix ***Q*** to vary at sites with and without coyotes:

$$Q_{\mathrm{wtd}|\text{coyote present}}=\left[-\exp\left(\mu_{11}\right)\;\exp\left(\mu_{11}\right)\;\exp\left(\mu_{12}\right)-\exp\left(\mu_{12}\right)\;\right],$$

$$Q_{\mathrm{wtd}|\text{coyote absent}}=\left[-\exp\left(\mu_{21}\right)\;\exp\left(\mu_{21}\right)\;\exp\left(\mu_{22}\right)-\exp\left(\mu_{22}\right)\;\right].$$

We then used rate parameters embedded in the matrices to calculate the proportion of time spent in the detectable state:

$$\pi_{\mathrm{wtd}|\text{coyote present}}=\frac{\exp\left(\mu_{11}\right)}{\exp\left(\mu_{11}\right)+\exp\left(\mu_{12}\right)},$$

$$\pi_{\mathrm{wtd}|\text{coyote absent}}=\frac{\exp\left(\mu_{21}\right)}{\exp\left(\mu_{21}\right)+\exp\left(\mu_{22}\right)}.$$

Finally, we evaluated if the proportion of time a white‐tailed deer spends in the detectable state differed at sites with and without coyotes by testing if the ratio $\pi_{\mathrm{wtd}|\text{coyote present}}/\pi_{\mathrm{wtd}|\text{coyote absent}}$ was different from 1.

We evaluated the detection intensity of white‐tailed deer by modeling parameter *λ* as a function of time of day (using a Fourier series basis function; Kellner et al., 2022) and NDVI:

$$\begin{array}{c} \lambda_{\mathrm{wtd}|\text{coyote present}}=\frac{\beta_{1}^{\mathrm{wtd}}}{2}+\beta_{2}^{\mathrm{wtd}}\cos\left(\frac{\mathrm{\pi t}}{12}\right)+\beta_{3}^{\mathrm{wtd}}\sin\left(\frac{\mathrm{\pi t}}{12}\right)\beta_{4}^{\mathrm{wtd}}\cos\left(\frac{2\mathrm{\pi t}}{12}\right)\\ +\beta_{5}^{\mathrm{wtd}}\sin\left(\frac{2\mathrm{\pi t}}{12}\right)+\beta_{6}^{\mathrm{wtd}}\text{NDVI}, \end{array}$$

$$\begin{array}{c} \lambda_{\mathrm{wtd}|\text{coyote absent}}=\frac{\beta_{7}^{\mathrm{wtd}}}{2}+\beta_{2}^{\mathrm{wtd}}\cos\left(\frac{\mathrm{\pi t}}{12}\right)+\beta_{3}^{\mathrm{wtd}}\sin\left(\frac{\mathrm{\pi t}}{12}\right)\beta_{4}^{\mathrm{wtd}}\cos\left(\frac{2\mathrm{\pi t}}{12}\right)\\ +\beta_{5}^{\mathrm{wtd}}\sin\left(\frac{2\mathrm{\pi t}}{12}\right)+\beta_{8}^{\mathrm{wtd}}\text{NDVI}. \end{array}$$

We evaluated if white‐tailed deer detection intensity differed at sites with and without coyotes by testing if $\beta_{1}^{\mathrm{wtd}}-\beta_{7}^{\mathrm{wtd}}$ (i.e., the intercept parameters for both regressions) was different from 0.

We fit models with a custom likelihood function written in the Stan modeling language (Stan Development Team,  2023). Note that while Stan is perhaps best known for Bayesian inference, we used it to optimize a likelihood function, since the run time for a fully Bayesian analysis was unreasonably long. Inference for this analysis was therefore based on maximum likelihood techniques. We used parametric bootstrapping to calculate the confidence intervals of derived parameters. All code used in the analysis is freely available at Zenodo (https://doi.org/10.5281/zenodo.8280379).

## 3 RESULTS

We obtained data from 319 camera trap deployments that cumulatively operated for 10,733 days or an average of 34 days per camera trap. From these deployments, 8943 sequences included photographs of white‐tailed deer and 173 sequences included photographs of coyotes. Across the seven study sites, naïve occupancy of white‐tailed deer ranged between 0.88 and 1.00, and naïve occupancy of coyotes ranged between 0.09 and 0.30 (Table A1).

The estimated marginal white‐tailed deer occupancy probability was 0.96 (95% CI = 0.92–0.98) and coyote occupancy probability was 0.47 (95% CI = 0.41–0.54). As indicated by an interaction coefficient *f*_3_ of 1.56 (95% CI = −0.86 to 3.82), white‐tailed deer were slightly more likely to occur at sites when coyotes were present, but 95% confidence intervals of this parameter overlapped 0, indicating that white‐tailed deer and coyotes occurred at sites independent of the other species.

When coyotes were present, the proportion of time that white‐tailed deer spent in the detectable state was 0.009 (95% CI = 0.008–0.011), which translates to approximately 13 min per day (95% CI = 11–16 min per day). When coyotes were absent, the proportion of time that white‐tailed deer spent in the detectable state was 0.004 (95% CI = 0.002–0.006), which translates to approximately 6 min per day (95% CI = 3–9 min per day). Thus, white‐tailed deer spent just over 2 times (95% CI = 1.3–4.3) more time in the detectable state when coyotes were present relative to when coyotes were absent. Since 95% confidence intervals of this ratio do not overlap 1, this indicates white‐tailed deer spent significantly more time in the detectable state when coyotes were present relative to when coyotes were absent. The proportion of time that coyotes spent in the detectable state was 0.002 (95% CI = 0.001–0.003), which translates to approximately 3 min per day (95% CI = 1–5 min per day).

**FIGURE 3:**
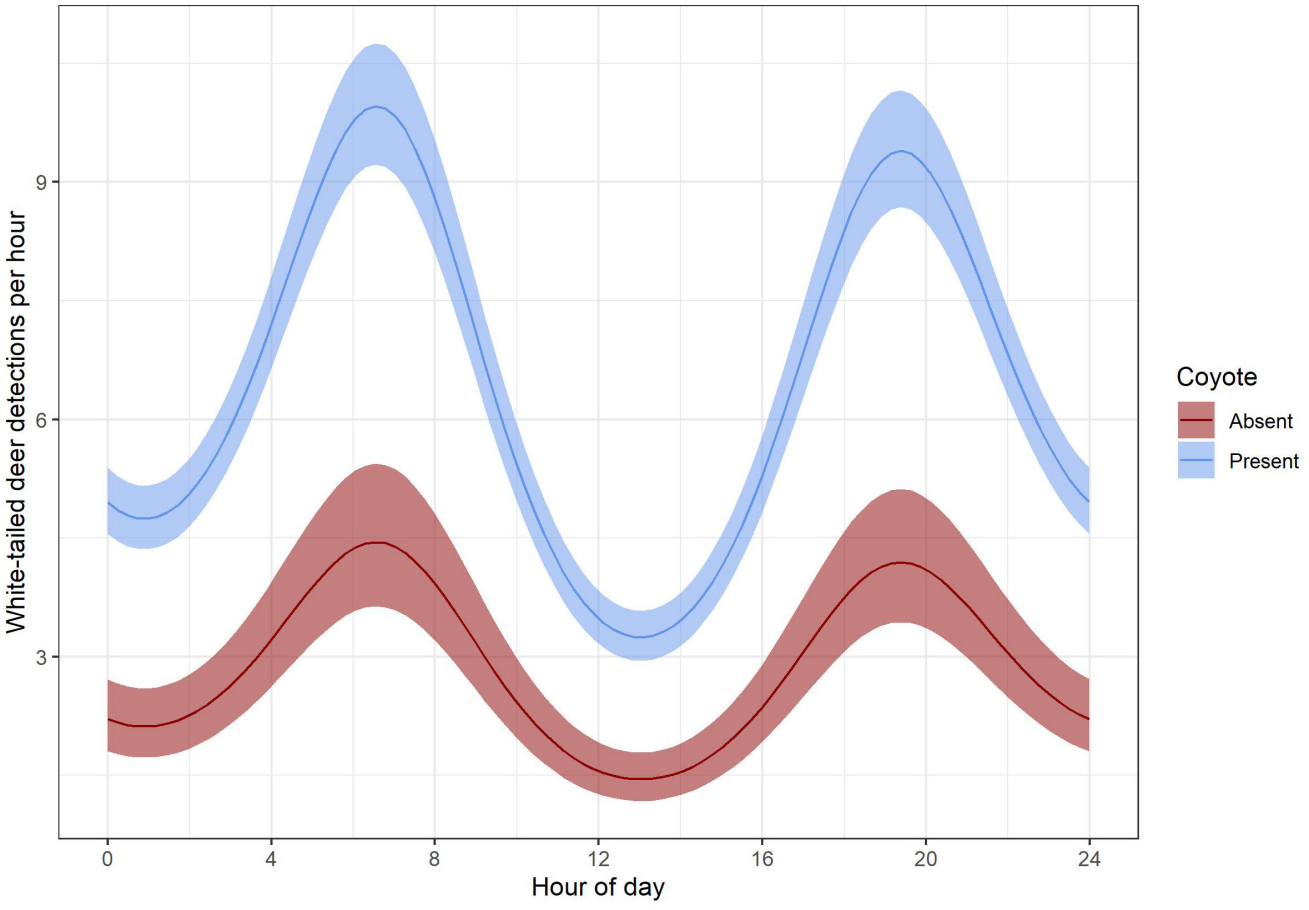
Detection intensity (number of detections per hour) of white‐tailed deer (*Odocoileus virginianus*) when coyotes (*Canis latrans*) are present and absent. This figure assumes the mean Normalized Difference Vegetation Index value observed. Data were obtained from camera traps deployed throughout the Central Appalachians region (West Virginia and Pennsylvania) of the eastern USA.

**FIGURE 4:**
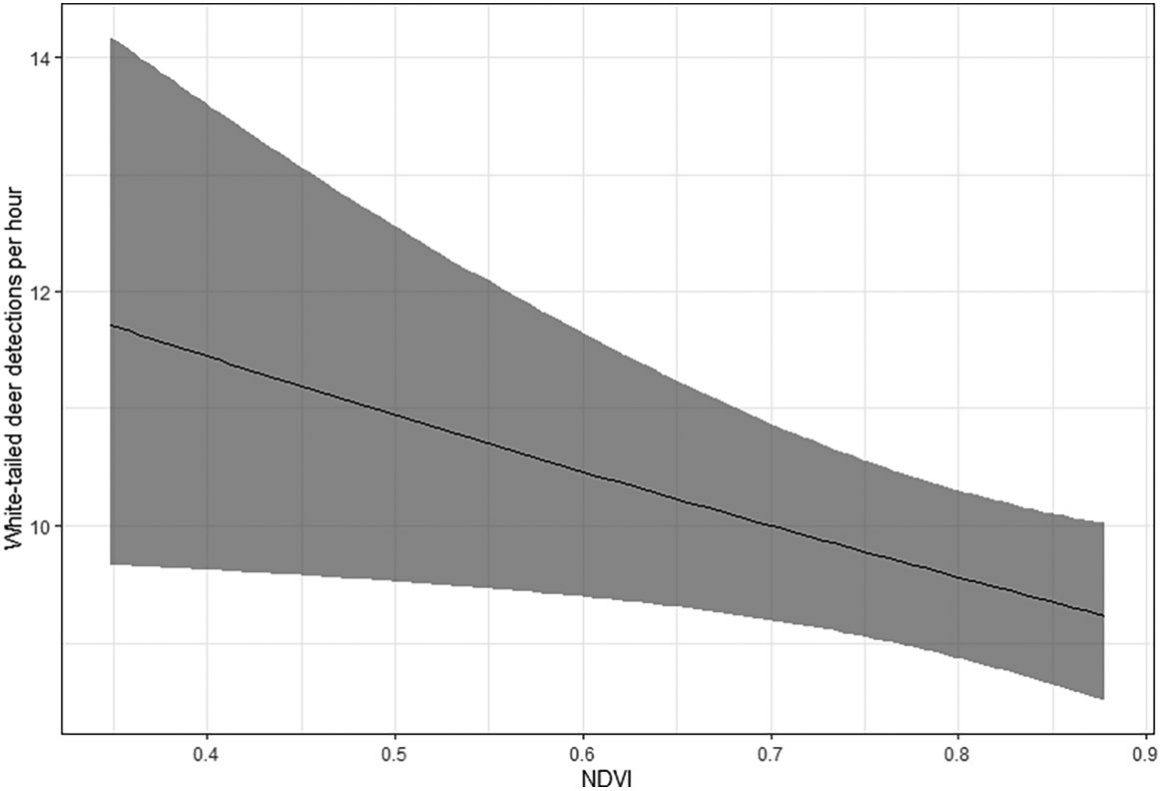
Detection intensity (number of detections per hour) of white‐tailed deer (*Odocoileus virginianus*) as a function of Normalized Difference Vegetation Index (NDVI) values when coyotes (*Canis latrans*) are present. This figure assumes detection intensity at 6 h after midnight. Data were obtained from camera traps deployed throughout the Central Appalachians region (West Virginia and Pennsylvania) of the eastern USA.

White‐tailed deer are largely crepuscular, and we correspondingly found detection intensity while in the detectable state was greatest around dawn and dusk (Figure 3). We also found that white‐tailed deer detection intensity was greater at sites with coyotes relative to sites without coyotes ($\beta_{1}^{\mathrm{wtd}}-\beta_{7}^{\mathrm{wtd}}$ = 1.6, 95% CI = 1.2–2.0). Since 95% confidence intervals of this difference do not overlap 0, this indicates white‐tailed deer detection intensity was significantly greater at sites with coyotes relative to sites without coyotes. Detection intensity of white‐tailed deer was negatively related to NDVI at sites where coyotes were present ($\beta_{6}^{\mathrm{wtd}}$ = −0.05, 95% CI = −0.09 to −0.01, Figure 4) but was not related to NDVI at sites where coyotes were absent ($\beta_{8}^{\mathrm{wtd}}$ = 0.02, 95% CI = −0.12 to 0.18). Coyotes are largely nocturnal, and we correspondingly found detection intensity while in the detectable state was greatest at night. A complete list of parameter estimates can be found in Table A2.

## 4 DISCUSSION

In our study, white‐tailed deer detection rates increased at sites where coyotes were present. Our results support the Active Response Hypothesis, which posits that white‐tailed deer would exhibit increased detection rates when coyotes were present because more movement, whether a reactive (e.g., fleeing) or proactive response, would decrease the probability of adverse encounters with coyotes, and/or because coyotes tended to occupy sites with higher white‐tailed deer densities. We found that white‐tailed deer and coyote occupancy probabilities were independent, but white‐tailed deer spent more time in the detectable state and had greater detection intensity at sites where coyotes were present, regardless of vegetation‐related covariates. Based on relationships with both time of day and NDVI, the presence/absence of coyotes did not affect the overall daily activity patterns of white‐tailed deer, but it did affect the influence of vegetation on detection intensity. At sites with no coyotes, NDVI was not related to white‐tailed deer detection intensity, but there was a significant relationship at sites with coyotes, such that white‐tailed deer detection intensity was highest when presumed forage availability was relatively low. Collectively, these findings suggest that white‐tailed deer may be actively altering their behavior (e.g., movement rates) in relation to predation risk, but they generally do not use temporal partitioning as their primary strategy for avoiding encounters with coyotes. Concurrently, coyote presence may be related to the density of white‐tailed deer populations, which underscores the complexity of predator–prey relationships in our study system.

As posited in the Active Response Hypothesis, one mechanism that could result in greater prey species detection rates in areas with predators is an increase in movement rates. The interplay between predator and prey movements is often thought of as a “response race” in which prey are constantly making movement decisions to avoid predators, while at the same time, predators are constantly making movement decisions to find and kill prey (Mitchell & Lima, 2002; Sih, 2005). Although prey species can use predictive behavioral responses (e.g., avoiding areas where predators occur frequently; Creel & Christianson, 2008), they can also rely on reactive responses (e.g., altering finer‐scale space use decisions in response to a more immediate risk; Courbin et al., 2016). For example, white‐tailed deer frequently engage in a fleeing response when encountering coyotes (Lingle & Pellis, 2002). Based on the occupancy patterns from our study, white‐tailed deer did not appear to avoid sites where coyotes were present, but their detection rates did increase at those sites, particularly when presumed forage availability (i.e., NDVI value) was relatively low (Figure 4). These observations support a positive relationship between detection rates and movement rates; thus, our results could be interpreted to align with the movement rate component of the Active Response Hypothesis. It is possible that white‐tailed deer may be exhibiting an active avoidance behavioral response to predators by increasing movement rates when coyotes are present in an area due to reactive evasive maneuvers (e.g., fleeing) and/or proactive attempts to avoid or reduce adverse encounters with them (e.g., predation events) (Frair et al., 2005).

Altering movement rates can be effective anti‐predator behaviors in various systems, but increasing movement rates might be the more profitable anti‐predator behavior in our study system for several reasons. First, reducing movement rates could come at an energetic cost if forage is not readily available in localized areas (Lima & Dill, 1990). Our study sites likely contain patchy high‐quality food resources for white‐tailed deer, so a reduction of movement rates would limit the amount of high‐quality forage available to individuals. This conjecture seems supported by the observation that white‐tailed deer detection rates were highest at sites with lower presumed forage availability. Second, increasing movement rates rather than remaining stationary is considered to be a better predator avoidance strategy when predators have good spatial memories (Mitchell & Lima, 2002). Previous research suggests that coyotes have strong spatial memories, as evidenced by their ability to find food caches several months after making them (O'Donoghue et al., 1997). Therefore, reducing movement rates and remaining in a localized position could increase the risk to white‐tailed deer from coyotes in this system.

Alternatively, or additionally, to movement rates, our observed result of increased white‐tailed deer detection rates at sites with coyotes could be driven by a relationship between coyote occupancy and white‐tailed deer population density. Although variation in study design and species characteristics can affect the correlation between species density and detection rates (Sollmann et al., 2013), white‐tailed deer densities are highly correlated with detection rates using camera trap study designs similar to our investigation (Parsons et al., 2017). If greater white‐tailed deer density led to increased white‐tailed deer detection rates when coyotes were present, our results could be indicative of an active response by coyotes. Predator species often have greater occupancy probabilities in areas with higher prey density (Everatt et al., 2019; Rabelo et al., 2019); thus, coyotes might have been preferentially selecting for areas with more white‐tailed deer.

Our results were similar to Kellner et al. (2022), who also used camera trap data to model temporal and spatial interactions between white‐tailed deer and coyotes. They modeled white‐tailed deer detection intensity as a function of time at sites with and without coyotes and found that white‐tailed deer detection rates were greater when coyotes were present, just as we did. However, we additionally incorporated a vegetation‐related covariate (i.e., NDVI) into our model, and our results pertaining to temporal patterns of white‐tailed deer in the presence of coyotes diverged from those of Kellner et al. (2022). In both studies, white‐tailed deer were largely crepuscular, whereas coyotes were largely nocturnal, with some overlap in their activity patterns, but we found that white‐tailed deer did not exhibit large shifts in daily activity patterns based on coyote occupancy (Figure 3). This finding suggests that white‐tailed deer are generally not exhibiting temporal partitioning behavior to avoid encounters with coyotes in our study region. It is possible that a temporal response would become evident if we had categorized our white‐tailed deer detections by age class and/or sex. Previous studies have documented that both fawns and maternal females with fawns display more diurnal activity patterns, likely because neonates are much more commonly preyed upon than adults (Crawford et al., 2021; Higdon et al., 2019; Jackson et al., 1972), although predation on adult female white‐tailed deer has also been documented (Campbell et al., 2005; Chitwood et al., 2014; Whitlaw et al., 1998). Due to the time of year when many of the camera traps were deployed, fawns were not always available to be detected or could not always be readily identified from photographs, limiting our ability to assess age‐specific responses. When considering the overall white‐tailed deer population in our study area, we conclude that any benefits gained from shifting to a more diurnal activity pattern to decrease temporal overlap with coyotes may not outweigh the costs (Beier & McCullough, 1990).

Some aspects of white‐tailed deer behavior that may influence detection rates in the presence of coyotes might be context dependent, such that we might expect to find variation in the observed patterns under different circumstances or within different regions. For example, we used data that were collected primarily in spring, summer, and fall, but we were unable to directly incorporate the potential effect of season on detection rates into our data analyses. Fawns are especially vulnerable to coyote predation (Kilgo et al., 2012), so we might expect stronger anti‐predator behavioral responses from females or fawns during the late spring and early summer period when predation risk from coyotes is highest. Similarly, male white‐tailed deer are most vulnerable to predation risk during the winter after the breeding season occurs (Gulsby et al., 2018); therefore, we might expect to see seasonal variation in detection rates of male white‐tailed deer in relation to predation risk. In addition, landscape context could be another factor influencing how white‐tailed deer respond to risk from coyotes. Habitat complexity can influence predator–prey dynamics in a variety of systems (Camp et al., 2012; Smith, Donadio, Pauli, Sheriff, Bidder, & Middleton, 2019). Specific to white‐tailed deer and coyotes, some previous research suggests that fawns are at a higher risk of predation in homogenous landscapes with fewer edges (Gulsby et al., 2017), whereas other studies did not find a strong effect of vegetation on fawn predation risk (Kilgo et al., 2014; Vreeland et al., 2004). Collecting year‐round data and incorporating age classes, season, and landscape‐level habitat covariates into our modeling approach could provide further insight into the effect of coyote presence on white‐tailed deer detection rates.

Although we found evidence in support of the Active Response Hypothesis, there are factors that we were not able to incorporate into our research which could have influenced our observed results. White‐tailed deer may alter space use for a variety of reasons, including foraging (Massé & Côté, 2013), mate acquisition (Buderman et al., 2021; Foley et al., 2015), avoiding humans (Little et al., 2016), and changes in weather conditions (Beier & McCullough, 1990). Therefore, we cannot ascribe our observed white‐tailed deer detection rates to predation risk from coyotes alone. In addition, white‐tailed deer might use behavioral strategies for reducing predation risk other than those included in this study. For example, white‐tailed deer have been found to change vigilance levels in response to increased predation risk (Gulsby et al., 2018; Lashley et al., 2014). Finally, although coyotes do represent a risk to both fawns (Kilgo et al., 2012) and adults (Chitwood et al., 2014), coyotes do not represent the majority of adult white‐tailed deer mortality in the eastern United States (Campbell et al., 2005). White‐tailed deer may exhibit stronger responses to humans (e.g., hunters) or larger natural predators, such as wolves (*Canis lupus*) or mountain lions (*Puma concolor*), which pose a greater threat to all age classes (Nelson & Mech, 1986; Veals Dutt et al., 2023). For example, a Michigan study found that maternal females and fawns selected against areas used by wolves, but did not similarly avoid coyotes, despite coyotes being the leading source of fawn predation (Duquette et al., 2014). It would thus be valuable to study variation in white‐tailed deer anti‐predator behaviors in ecosystems with larger predators to further build upon our understanding of how white‐tailed deer respond behaviorally to predation risk.

Ultimately, our study implements a recently developed analytical approach using continuous‐time multi‐species occupancy models to gain novel ecological insight into the complex relationships between predator and prey species. We encourage the use of this methodology to investigate a myriad of ecological processes, including predator–prey dynamics (Smith et al., 2020; Suraci et al., 2022), and answer questions related to the spatial and temporal interactions of multiple species while accounting for imperfect detection. Expanding upon this investigation, additional research on different pairs of species in different ecosystems across the world would provide insight into the context‐dependency of predator–prey interactions on a global scale.

## AUTHOR CONTRIBUTIONS

**Hannah L. Clipp:** Conceptualization (equal); data curation (equal); investigation (equal); visualization (equal); writing – original draft (equal); writing – review and editing (equal). **Sarah M. Pesi:** Data curation (equal); investigation (equal); writing – original draft (equal); writing – review and editing (equal). **Madison L. Miller:** Data curation (equal); writing – original draft (equal); writing – review and editing (equal). **Laura C. Gigliotti:** Conceptualization (equal); writing – original draft (equal); writing – review and editing (equal). **Brett P. Skelly:** Resources (equal); writing – original draft (equal); writing – review and editing (equal). **Christopher T. Rota:** Conceptualization (equal); formal analysis (equal); funding acquisition (equal); methodology (equal); resources (equal); software (equal); supervision (equal); visualization (equal); writing – original draft (equal); writing – review and editing (equal).

## CONFLICT OF INTEREST STATEMENT

The authors of this article have no competing interests that would impact the integrity of this work.

## Data Availability

All of the camera trap data used in this investigation are archived at Wildlife Insights (https://www.wildlifeinsights.org/). Processed species occurrence data from the camera traps and Program R code associated with this study are freely available in a Zenodo repository (https://doi.org/10.5281/zenodo.8280379).

## Appendix

**TABLE A1:** Summary of naive occupancy of coyotes (*Canis latrans*) and white‐tailed deer (*Odocoileus virginianus*) in our study, arranged by species and study area/study site.

Study area/study site	Naive coyote occupancy	Naive deer occupancy
Monongahela National Forest, WV	0.11	0.88
Private lands, WV	0.30	0.94
University Research Forest, WV	0.09	0.97
Rothrock State Forest, PA	0.17	1.00
Gettysburg National Military Park, PA	0.29	0.86

*Note*: See Figure 2 for the mapped locations of all study areas and study sites in West Virginia (WV) and Pennsylvania (PA).

**TABLE A2:** Coefficient estimates and their standard errors from a two‐species occupancy model with continuous‐time detection processes that tested for spatial and temporal interactions between white‐tailed deer (wtd, *Odocoileus virginianus*) and coyotes (coy, *Canis latrans*).

Coefficient	Estimate	Standard error	*z*	*p*
$\mu_{11}$	−2.13	0.03	−82.75	.00
$\mu_{21}$	−3.05	0.03	−91.47	.00
$\mu_{31}$	−4.32	0.07	−62.69	.00
$\mu_{12}$	2.55	0.08	30.75	.00
$\mu_{22}$	2.52	0.28	9.07	.00
$\mu_{32}$	2.05	0.32	6.35	.00
$\beta_{1}^{\mathrm{wtd}}$	3.63	0.06	56.16	.00
$\beta_{2}^{\mathrm{wtd}}$	0.18	0.02	7.87	.00
$\beta_{3}^{\mathrm{wtd}}$	0.08	0.02	4.00	.00
$\beta_{4}^{\mathrm{wtd}}$	−0.39	0.02	−19.22	.00
$\beta_{5}^{\mathrm{wtd}}$	−0.22	0.02	−10.25	.00
$\beta_{6}^{\mathrm{wtd}}$	−0.05	0.02	−2.22	.03
$\beta_{7}^{\mathrm{wtd}}$	2.01	0.20	10.07	.00
$\beta_{8}^{\mathrm{wtd}}$	0.02	0.08	0.30	.76
$\beta_{1}^{\mathrm{coy}}$	−0.49	0.76	−0.64	.52
$\beta_{2}^{\mathrm{coy}}$	0.22	0.12	1.84	.07
$\beta_{3}^{\mathrm{coy}}$	0.11	0.13	0.89	.37
$\beta_{4}^{\mathrm{coy}}$	0.11	0.12	0.90	.37
$\beta_{5}^{\mathrm{coy}}$	0.17	0.12	1.44	.15
$\beta_{5}^{\mathrm{coy}}$	−0.01	0.11	−0.05	.96
$f_{1}$	2.90	0.43	6.82	.00
$f_{2}$	−1.62	1.18	−1.38	.17
$f_{3}$	1.56	1.19	1.31	.19
